# Non-invasive diagnostics of blockage growth in the descending aorta-computational approach

**DOI:** 10.1007/s11517-022-02665-2

**Published:** 2022-09-27

**Authors:** Mohammad AL-Rawi, Ahmed M. AL-Jumaily, Djelloul Belkacemi

**Affiliations:** 1 Center for Engineering and Industrial Design (CEID), Waikato Institute of Technology (Wintec), Hamilton, New Zealand; 2 Institute of Biomedical Technologies (IBTec), Auckland University of Technology (AUT), Auckland, New Zealand; 3 Mechanics and Energetics Laboratory, Hassiba Ben Bouali University Chlef, Ouled Fares, Algeria

**Keywords:** Atherosclerosis, Oscillatory shear index, Relative residence time, Carreau-Yasuda model, CFD

## Abstract

Atherosclerosis causes blockages to the main arteries such as the aorta preventing blood flow from delivering oxygen to the organs. Non-invasive diagnosis of these blockages is difficult, particularly in primary healthcare. In this paper, the effect of arterial blockage development and growth is investigated at the descending aorta on some possible non-invasive assessment parameters including the blood pressure waveform, wall shear stress (WSS), time-average WSS (TAWSS) and the oscillation shear index (OSI). Blockage severity growth is introduced in a simulation model as 25%, 35%, 50% and 65% stenosis at the descending aorta based on specific healthy control aorta data clinically obtained. A 3D aorta model with invasive pulsatile waveforms (blood flow and pressure) is used in the CFD simulation. Blockage severity is assessed by using blood pressure measurements at the left subclavian artery. An arterial blockage growth more than 35% of the lumen diameter significantly affects the pressure. A strong correlation is also observed between the ascending aorta pressure values, pressure at the left subclavian artery and the relative residence time (RRT). An increase of RRT downstream from the stenosis indicates a 35% stenosis at the descending aorta which results in high systolic and diastolic pressure readings. The findings of this study could be further extended by transferring the waveform reading from the left subclavian artery to the brachial artery.

**Graphical abstract:**

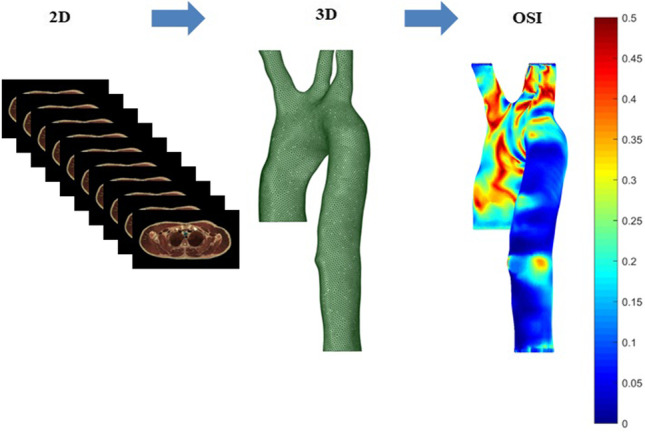

## Introduction

In recent years, advances in computational modelling techniques enabled biomedical researchers’ insight into the cardiovascular system using these techniques such as computational fluid dynamics (CFD) which facilitates investigation of various cardiovascular diseases including atherosclerosis and aneurysm. Several studies assumed different rheological properties to simulate the blood models numerically [1–5]. For example, blood can be simulated assuming Newtonian properties for large arteries and non-Newtonian properties for small arteries. Typically, these studies present a patient-specific case to investigate the rheological properties and hemodynamic parameters for the blood flow in transient analysis. Other studies used CFD to examine the wall shear stress (WSS) and pressure (P) distribution for patients with aneurysms in various regions of the aorta [1–5]. They found that the arteries must be investigated in conjunction with the shear stress parameters such as the time-averaged wall shear stress (TAWSS) and the oscillation shear index (OSI). These parameters are obtained by calculating the WSS vector within a cardiac cycle.

Alternatively, Bampi et al. [6] used a different method to diagnose non-invasively coronary atherosclerosis (< 29% stenosis) on one hundred mixed-gender patients based on comparing clinical and laboratory data to determine and predict the extent of the stenosis using high-resolution ultrasound. The non-invasive prediction was based on the calcium score and presence of LDL cholesterol along with the ratio of TG/HDL-c.

Atherosclerosis originated from small injuries to the blood vessel wall, forming a thickening wound at the injury location of the wall which has also been investigated. This local thickening is self-reinforcing: gradually more material accumulates at the location leading to arterial stenosis [7, 8]. These deposits, called arterial plaque, are composed of calcium, platelets, cellulose material, fats and cholesterol [9–11]. Dabagh et al. [12] studied three stages of atherosclerosis plaque growth at the branches above the aortic arch to correlate between the WSS contours and the plaque size, assuming uniform Newtonian blood properties. They found that the value of WSS exceeds 50 Pa values at the location with 80% stenosis severity on both the left common carotid and brachiocephalic arteries. Chen and Lu [13] and Kumar et al. [14] analysed, in a bifurcation model, the impact of assuming Newtonian and non-Newtonian blood properties, to identify appropriate governing equations to reflect shear-thinning behaviour. They found that the Carreau-Yasuda model plays a significant role in addressing the hemodynamic parameters and rheological properties such as WSS in arteries.

Kamangar et al. [15] investigated the impact of stenosis development on the hemodynamic parameters such as WSS in the course of hyperemic flow condition developed on a 3D artery geometries created based on CT images. The results indicated that high WSS vectors are attributed to the percentage of the area of stenosis combined with a recirculation zone after the injury location. They have also found that this recirculation zone exacerbates the development of stenosis. Bit et al. [16] analysed a 3D patient-specific aorta geometry numerically with aortic arch stenoses in the descending aorta assuming non-Newtonian blood properties (power-Law model). They found that 25% of stenosed artery contributes to the change of the following parameters such as the oscillation shear index (OSI) and relative residence time (RRT) in the ascending aorta region. Due to the proportionality between RRT values and the time blood particles spend near the artery wall (the transient time), we will include RRT in our study.

Fytanidis et al. [17] examined the relation between WSS, TAWSS, OSI and RRT to address the atherosclerosis development in the aortic region numerically. Low WSS and high OSI were found to cause a thickening of the wall in a healthy aorta, as well as the inferior and anterior walls of the brachiocephalic artery. They also found that RRT attains high values in areas with low WSS vectors, which may identify where atherosclerosis can develop and grow in specific aortic regions. Soulis et al. [18] compared numerically non-Newtonian models to a Newtonian model for a healthy human aorta to investigate the TAWSS, OSI and RRT. They found that high RRT contours developed in the aortic arch after the left subclavian artery. Also, using non-Newtonian properties shows an elevation in the RRT contours. High RRT distribution with low WSS and high OSI appears to indicate possible locations of atheromatic plaque with a reasonable degree of accuracy. Pandey et al. [19] concluded numerically that treating the blood as non-Newtonian obtains an acceptable approximation to the WSS results when investigating causation and formation of arterial plaque. They also observed that high OSI at the bifurcations suggests there is a region of low WSS with oscillatory properties. Malota et al. [20] investigated stenosis computationally on the coronary artery assuming Newtonian blood properties. They found that the spatial and sequential fluctuations of the WSS contours determined the mean of OSI and RRT, where 30–40% stenosis is shown by a linear increase to the OSI and a high degree of stenosis, 40–60%, will increase the OSI and RRT values to the maximum. Azar et al. [21] used CFD tools to investigate severe carotid artery stenosis using non-Newtonian Carreau blood properties. They found that the RRT contours were negatively correlated to the stenosis severity.

The aforementioned literature [1–6, 22, 23] identifies the following key mechanical aspects:

1. The frictional force applied to the endothelium by the blood mass flow rate is characterised by the WSS contour profile.
2. Atherosclerosis develops more readily in areas displaying low TAWSS values.
3. The OSI can be used to identify locations where blood flow is recirculating as it measures differences in the WSS impact (direction) in a cardiac cycle.
4. The RRT is a key indicator of the likelihood of stenosis developing.

This paper investigates how the changes in the pressure waveform, WSS, TAWSS, OSI and RRT progress with the development of arterial blockages at the descending aorta. Severities of 25%, 35%, 50% and 65% are compared with the healthy aorta. In particular, this paper uses clinical data as boundary conditions to determine the changes in the hemodynamic parameters using CFD models. In addition, the research aims to develop a novel method which will diagnose the presence of atherosclerotic formations using the hemodynamic parameters: TAWSS, OSI, RRT and the severity of the stenosis.

## Methodology

In this section, we demonstrate the process of converting 2D images to a 3D aorta model using Materialize Mimics. We also analyse computationally the realistic boundary conditions obtained from a healthy patient with their CFD validation. A mesh independency for both the healthy (control) and four unhealthy aorta models with their governing equations embedded in ANSYS – Fluent® 2020R2 are considered in this study.

### Geometry reconstruction

Due to the non-thrombogenic branching element of aorta, modelling the healthy state of blood flow in this artery is a complex process [9, 12, 13]. Furthermore, oxygen transportation from the heart via arteries involves much higher physiologic pressure values compared to other vessels in the human body [9, 12, 13], which must be accounted for while generating the initial and final boundary conditions for the CFD simulation.

**Fig. 1:**
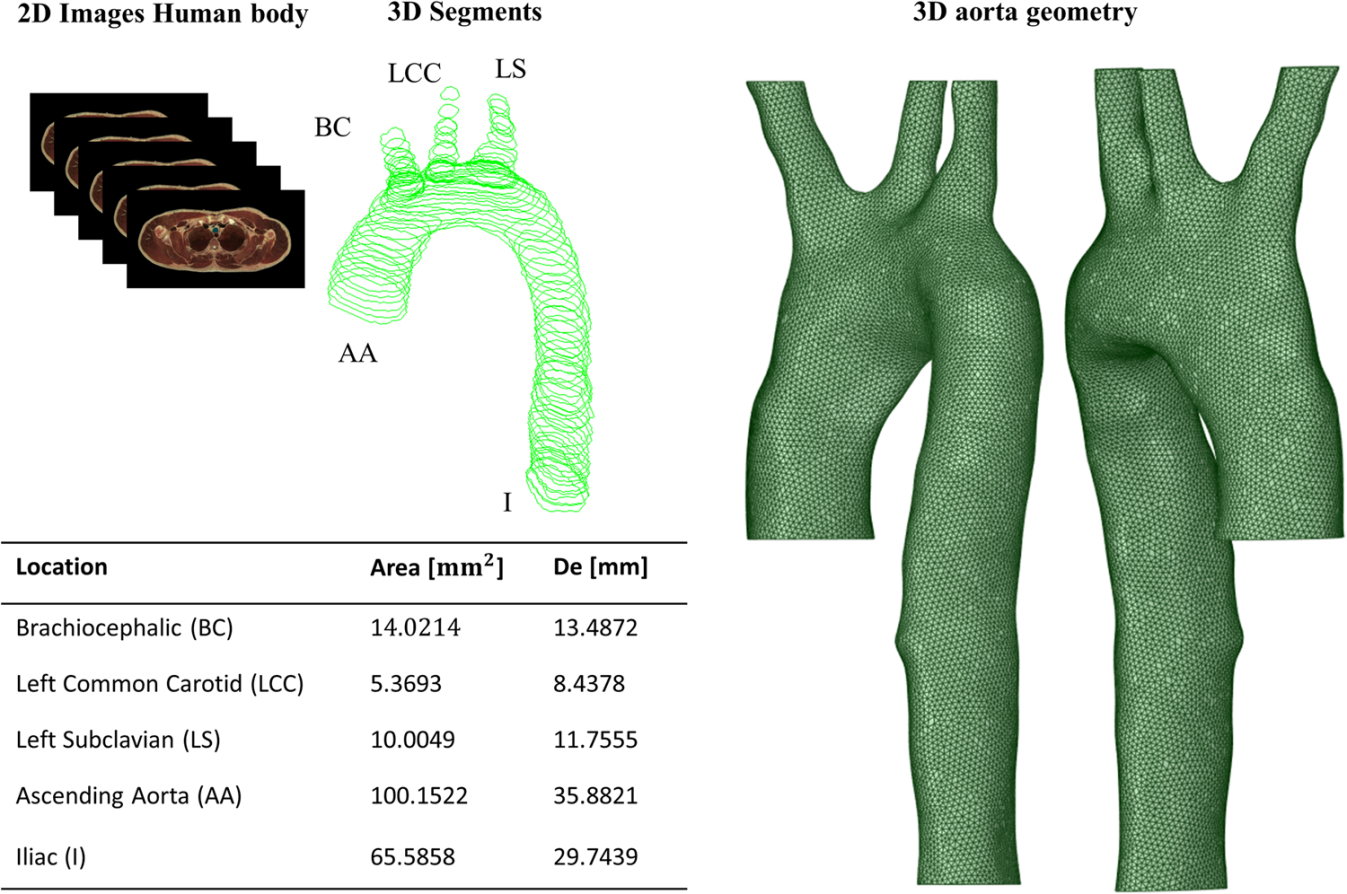
The process of converting 2D images to 3D aorta model using Materialize Mimics and SpaceClaim® 2020R2, also showing the dimensions for the inlet and four outlets of the aorta

To generate a 3D aorta geometry, we converted 2D images from CT scan to segments using Materialize Mimics, then exported them as an STL file to SpaceClaim® 2020R2 for refining the geometry and merging the faces of the aorta geometry as shown in Fig. 1. The SpaceClaim® 2020R2 tools are available under ANSYS®2020R2 and allow adjustment and configuration to the aorta including a removal of features to simplify the geometry. The 3D aorta geometry models include the three aortic arch branches with realistic orientation based on the CT images as shown in Fig. 1. While simplified assumptions were used in prior studies [18, 24–26], these resulted in the model differing to some degree from clinical trial data. Therefore, this study models a realistic healthy aorta, incorporating the behaviour of the three main aortic branches, as shown in Fig. 1.

**Fig. 2:**
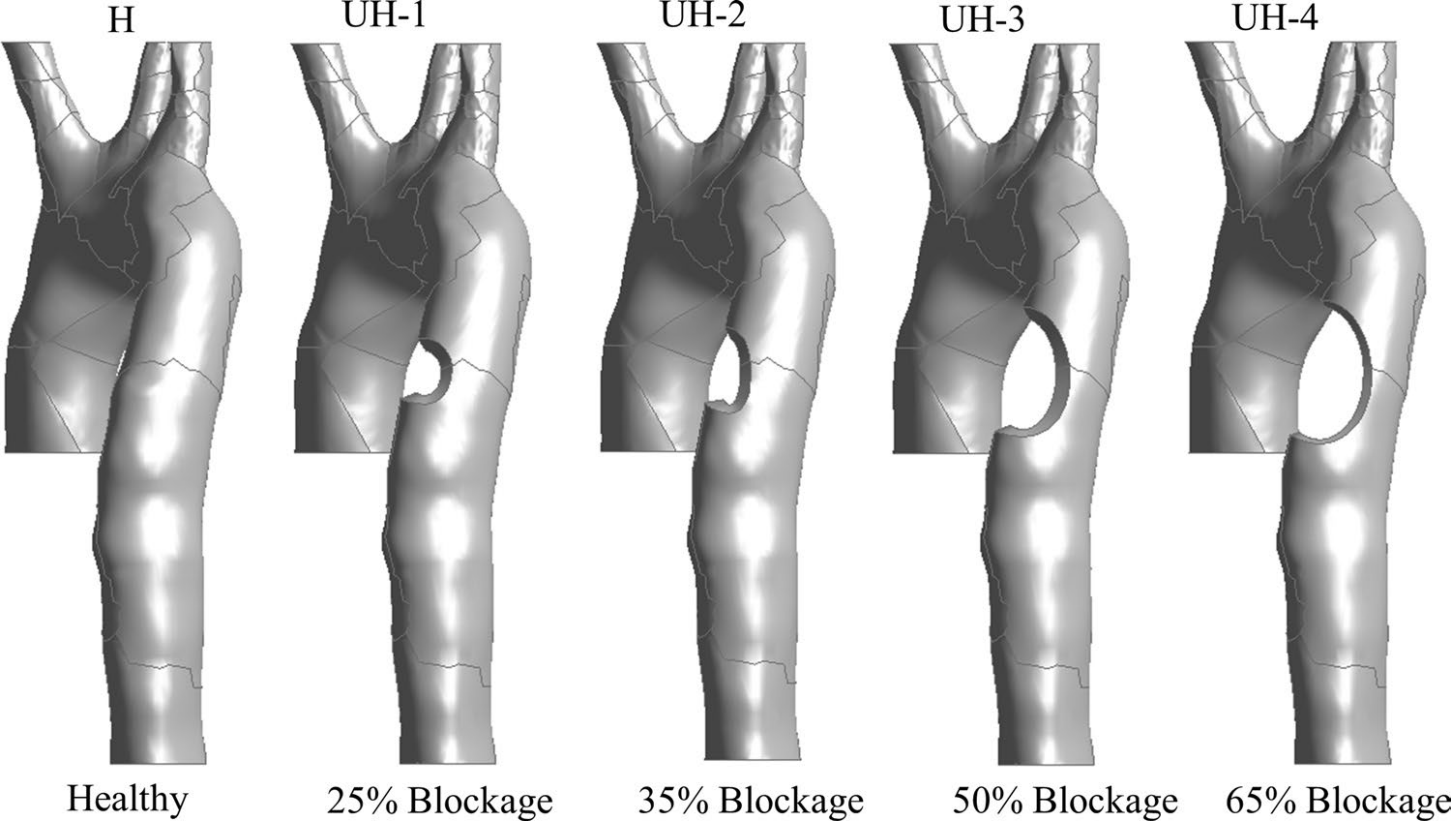
The healthy model and four unhealthy models with descending blockages (25%, 35%, 50% and 65% stenosis)

Based on the healthy (control) model shown in Fig. 1 and using SpaceClaim® 2020R2, we reconstructed four unhealthy models with an arterial blockage growth at the descending aorta based on a clinical study performed on rats [5]. These blockages represent different stages of the disease, moderate to severe with 25%, 35%, 50% and 65% stenosis (Fig. 2). The stenosis length ranged between 2 and 6 mm and the axial position ranged between 1.5 and 4 mm. Using the assessment parameters WSS, AWSS, TAWSS, OSI and RRT, in this study, we aim to correlate the stages of arterial blockage development by implementing non-Newtonian blood properties using the Carreau-Yasuda model and compare this to the control aorta model supported by the literature [12].

### Mesh generation and sensitivity

**Fig. 3:**
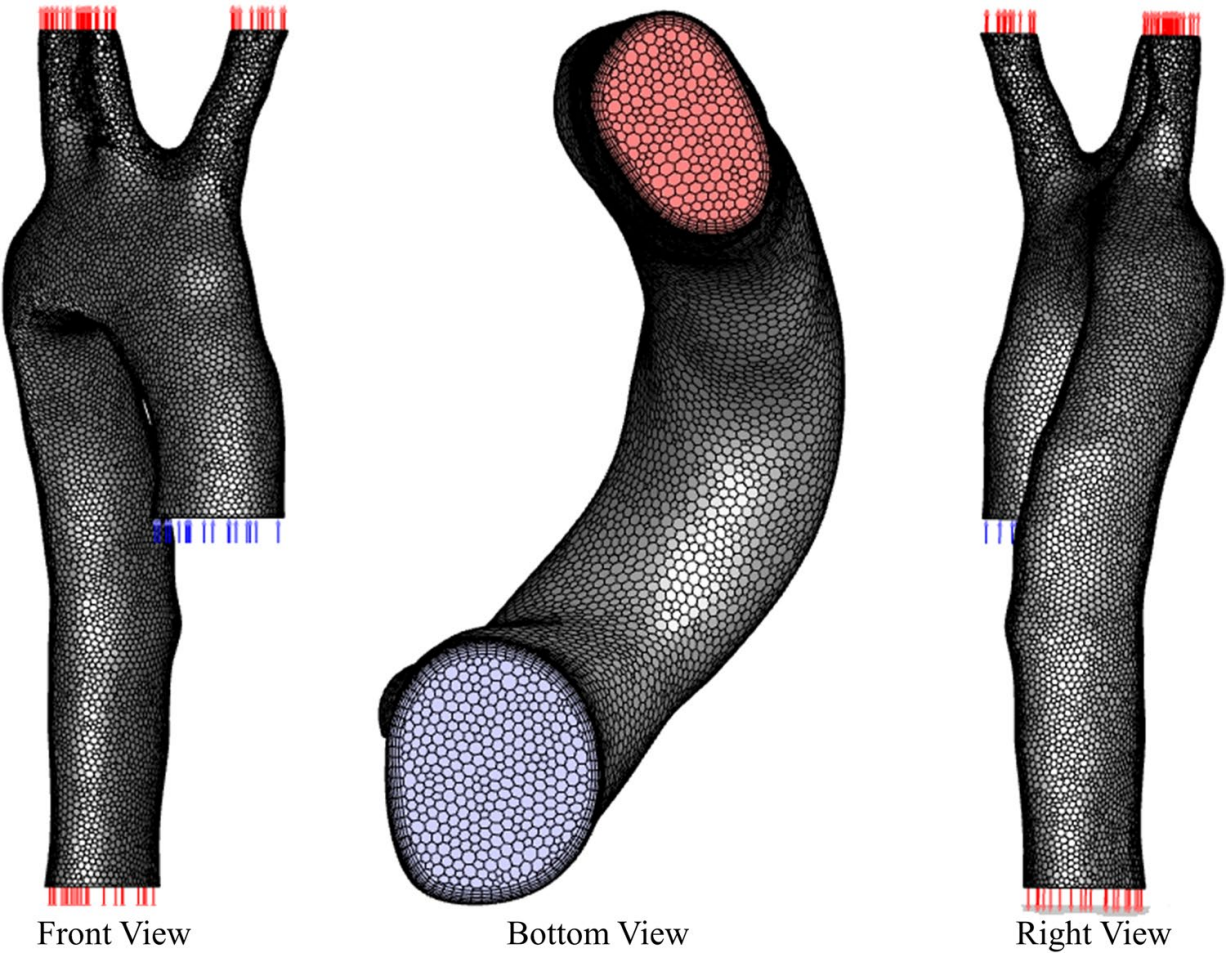
Polyhedral mesh for the healthy aorta with different views using Fluent

Mesh generation of the aorta models was performed using the tetrahedral method in ANSYS Workbench then converted this to a polyhedral mesh under ANSYS Fluent. In order to generate the model of the healthy aorta, we started with a simple mesh, then iteratively increased the number of elements until the optimal mesh size was identified. Table 1 shows the elements’ and nodes’ values for the healthy model with respect to systolic and diastolic pressure values and its sensitivity test. We found that the 0.25 mm element size with the inflation method, applied with 12 layers and a growth rate of 1.2 as the boundary mesh layer, achieves fine mesh independency, as shown in Fig. 3. In ANSYS®, “mesh metrics” enable examination and refinement of the mesh element quality to improve the simulation performance. ANSYS® 2020R2 includes a skewness measure, and this was evaluated in the present model, which had skewness measures from 0.71 to 0.77, which were within the acceptable range [5]. Furthermore, the chosen mesh had an acceptable numerical measure for moving meshing nodes and the convergences, in accordance with the literature [1, 4, 12]. The iteration maximum was set to achieve a reduction of relative and absolute residence times to reach convergence. This meant that iterations continued in anticipation of the convergence reaching the acceptable level, achieving the results which are shown in Table 1 [1, 2, 16, 22].

**Table 1:** Mesh sensitivity test results showing the systolic and diastolic pressure values for different mesh elements and nodes of the aorta control model [1, 2, 16, 22]

Geometry	Elements	Nodes	Systolic pressure (mmHg)	Difference (%)	Diastolic pressure (mmHg)	Difference (%)
Coarse mesh	2,568,714	565,831	138.21	7.172	67.76	3.276
Medium mesh	3,984,501	750,672	128.96	2.397	65.61	2.579
Fine mesh	4,953,950	1,132,850	125.94	-	63.96	-

Based on the mesh values for the control model, we have set the number of elements as shown in Table 2 for the four unhealthy models with increasing degrees of blockage and checked the skewness values to ensure they are in the acceptable range within the literature [1, 4, 12].

**Table 2:** The healthy and unhealthy mesh elements, nodes and skewness values for the aorta models

Model	Blockage	Elements	Nodes	Skewness
Healthy		4,953,950	1,132,850	0.72
Unhealthy	25%	4,927,739	1,129,799	0.71
	35%	4,917,144	1,127,404	0.76
	50%	4,760,642	1,097,976	0.77
	65%	4,661,954	1,080,363	0.76

### Mathematical modelling and boundary conditions

For both healthy and unhealthy models, the blood flow is assumed to be unsteady with pulsatile flow forms (laminar transition to turbulent), where the blood body is solved using Navier–Stokes equations as an adiabatic, incompressible fluid, with non-Newtonian blood properties. The aorta geometry is modelled with no-slip boundary conditions and wall rigidity, as per previous literature [27, 28]. The models are executed based on the equations set up into ANSYS®: (i) the equation of mass continuity (1) and (ii) the momentum Eq. (2) taking into account the stress tensor.

1 $$\frac{\partial \rho }{\partial t}+\nabla .\left(\rho \overrightarrow{v}\right)=0$$

2 $$\frac{\partial }{\partial t}\left(\rho \overrightarrow{v}\right)+\nabla .\left(\rho \overrightarrow{v}\overrightarrow{v}\right)=-\nabla p+\nabla .\overrightarrow{\tau }+\rho \overrightarrow{g}+\overrightarrow{F}$$

3 $$\overrightarrow{\tau }=\mu \left[\left(\nabla \overrightarrow{v}+\nabla {\overrightarrow{v}}^{T}\right)-\frac{2}{3}\nabla .\overrightarrow{v}I\right]$$

where $\rho$ is the blood density; $\frac{\partial \rho }{\partial t}$ reflects incompressible and transient pulsatile waveforms for the blood; and $\nabla .$ represents the divergence of $\overrightarrow{v}$ (which presents the velocity vector). Furthermore, the static pressure is given by $p$ in Eq. (2), such that $\nabla p$ gives the divergence of that pressure; and $\overrightarrow{\tau }$ represents the stress tensor, which is provided in Eq. (3). The terms $\overrightarrow{F}$ and $\uprho \overrightarrow{\mathrm{g}}$ describe, respectively, the external and gravitational forces exerted on the body. Lastly, the molecular viscosity is given by $\mu ;$ the unit tensor is $I;$ and the impact of volume dilation is described by the term $[\left(\nabla \overrightarrow{v}+\nabla {\overrightarrow{v}}^{T}\right)-\frac{2}{3}\nabla .\overrightarrow{v}I]$.

Employing a Newtonian blood flow assumption has good predictive power at high/advanced levels of atherosclerosis, or medium to high strain rates [18, 19]; however, the non-Newtonian assumption works better for lower strain rates [18, 19], which are of interest to our study. The target of our model is to non-invasively predict the early development of arterial blockages. Therefore, the blood properties are as follows: 1060 kg/m^3^ (density); non-Newtonian (viscosity); Carreau-Yasuda (shear-thinning) [29] as per

4 $$\mu ={\mu }_{\infty }+\frac{({\mu }_{o}-{\mu }_{\infty })}{({{1+(\lambda \dot{\gamma )}}^{a})}^{\frac{1-n}{a}}}$$

where the zero-shear viscosity ${\mu }_{o}=0.056$ $(\frac{kg}{m.s})$ and the infinite-shear viscosity ${\mu }_{\infty }=0.0035(\frac{kg}{m.s})$; the shear strain rate is given by $\dot{\gamma }$, while $\lambda =3.313$ (s) is the time constant and $n=0.3568$ is the Power-Law Index [29].

**Fig. 4:**
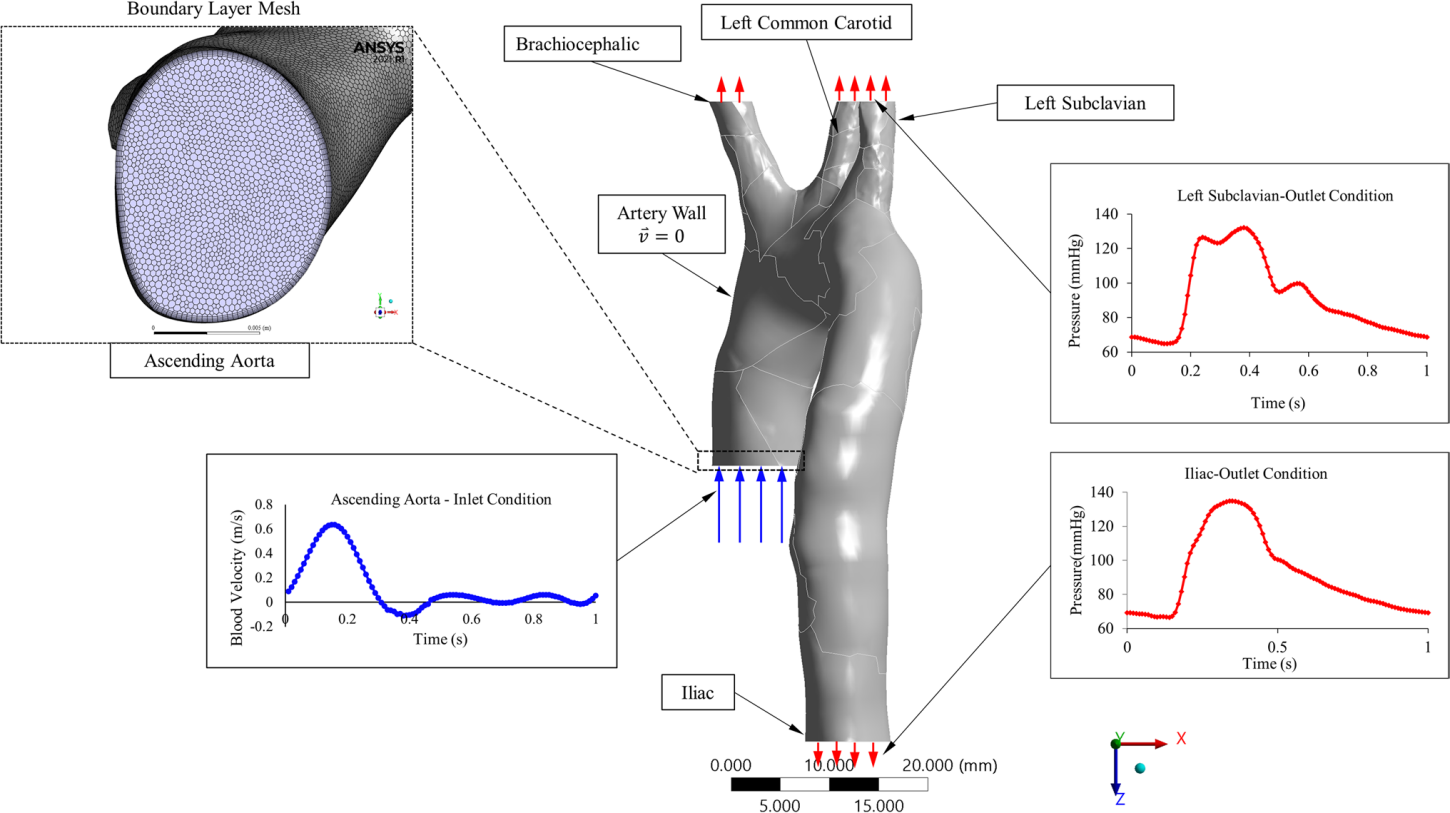
Boundary conditions: (**a**) the inlet blood flow waveform at the ascending aorta; (**b**) pressure waveform at the left subclavian artery; and (**c**) the pressure waveform at iliac artery

The boundary conditions are also set for the five aorta models assuming fixed (non-moving) inlet and outlet boundary conditions where the former is a blood flow velocity waveform [7, 29]. The three outlets’ boundary conditions above the aortic arch consisted of a left subclavian pressure waveform. The fourth outlet is set as an iliac pressure waveform. All waveforms were obtained clinically, as shown in Fig. 4. The pressure waveforms were invasively measured in a 40-year-old male undergoing left heart catheterization as approved using ethical approval NTX/09/11/109. Femoral and radial arterial access were used to introduce a fluid-filled catheter. Calibration of the manometer was performed as per the catheter laboratory’s standard protocol and checked to be within 1 mmHg of the calibrated non-invasive pressure sensor. The patient was awake and breathing spontaneously. The clinical data were recorded simultaneously using a Pulsecor R6.5B device during oscillometric blood pressure (left arm) measurements with the catheter at the ascending aorta (coronary sinus) and aortic arch (left subclavian take-off). The measurements were collected for 15-min durations, either towards the start or end of the routine investigation.

For the five geometries (one healthy and four with gradual blockage development) following the numerical simulation tools embedded in Fluent, we set up the model following these steps: (i) we generated a steady-state solution to form the initial boundary condition for the time-dependent solution. (ii) We defined the transient boundary conditions, as shown in Fig. 4, using the clinical data (inlet—velocity waveform and the outlets set to pulsatile pressure waveforms). (iii) The aorta wall was assumed stationary, and the shear condition set to no slip. (iv) Before running the results, we initialized the model using the hybrid initialization method. (v) Then, we calculated the transient solution (time step size 0.01 s) using the second-order implicit unsteady formulation and the coupled implicit solver. (vi) We undertook saving and post-processing time-dependent data sets in MATLAB.

## Results

In this study, a 3D healthy (control) aorta model with realistic boundary conditions’ waveforms (inlet and outlets’ velocity and pressure) is applied. The arterial blockage growth is investigated to determine the relationship between WSS, TAWSS, OSI and RRT for both healthy (control) and unhealthy models with descending blockage development from moderate to severe (UH-1 = 25%, UH-2 = 35%, UH-3 = 50% and UH-4 = 65% stenosis).

The transient analysis is performed for a 0.01 s time step for one consecutive cardiac cycle with a 70 bpm heart rate [30]. Within each time step, the stop criterion retains the same normalized change for each transferred value. Although a 1-D model is less computationally intensive, advances in processor power across the last decade have dramatically reduced the duration of simulations. This simulation took approximately 24 h per model, using Intel® Core™ i7-8750H CPU @ 2.21 GHz and 31.9 GB usable, 64-bit Operating System.

**Fig. 5:**
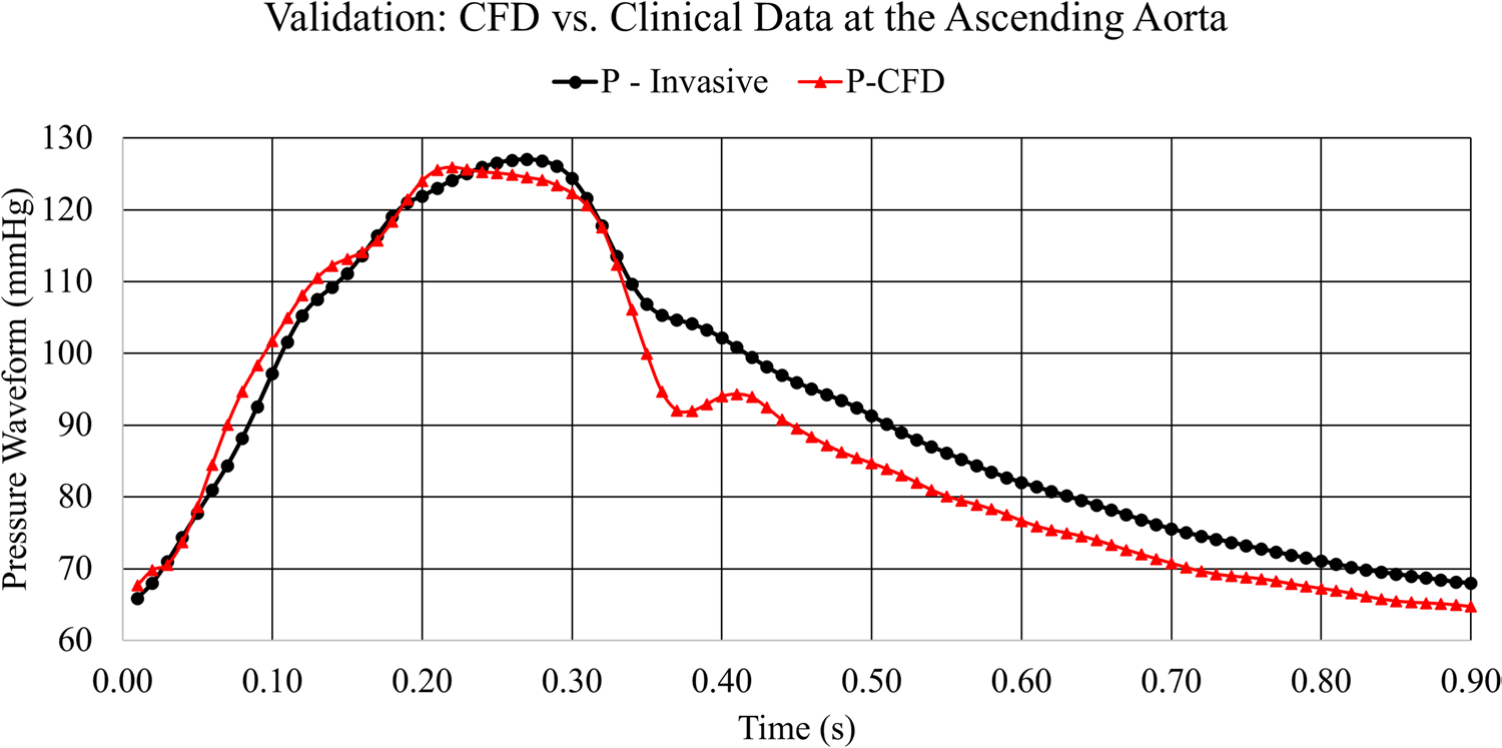
The CFD validation for the healthy aorta against the clinical data obtained at the ascending aorta

The validation of the healthy (control) model shows a very good agreement between the ascending pressure waveform measured clinically and the CFD simulated waveform generated from setting our boundary conditions as shown in Fig. 5. These two waveforms exhibit good agreement with clinical data, having an average error of 3.11% which is related to the iteration and time discretization errors. A high level of agreement is achieved due to employing realistic material properties via the Carreau-Yasuda model, and the clinically derived boundary conditions.

### Blood flow waveforms

The mechanisms by which the human body physiologically regulates its blood flow and exerts circulatory control can be explored via assessing the blood volume flow rate waveforms and pulsatile pressure waveforms in the vessels, allowing the modelling of healthy and unhealthy arteries [31–35]. In the case of atherosclerosis, we are particularly interested in identifying significant changes to blood flow behaviour in terms of patterns around the region of the disease development and growth, as these can be indicative of the presence of atherosclerosis at these locations [34, 35].

**Fig. 6:**
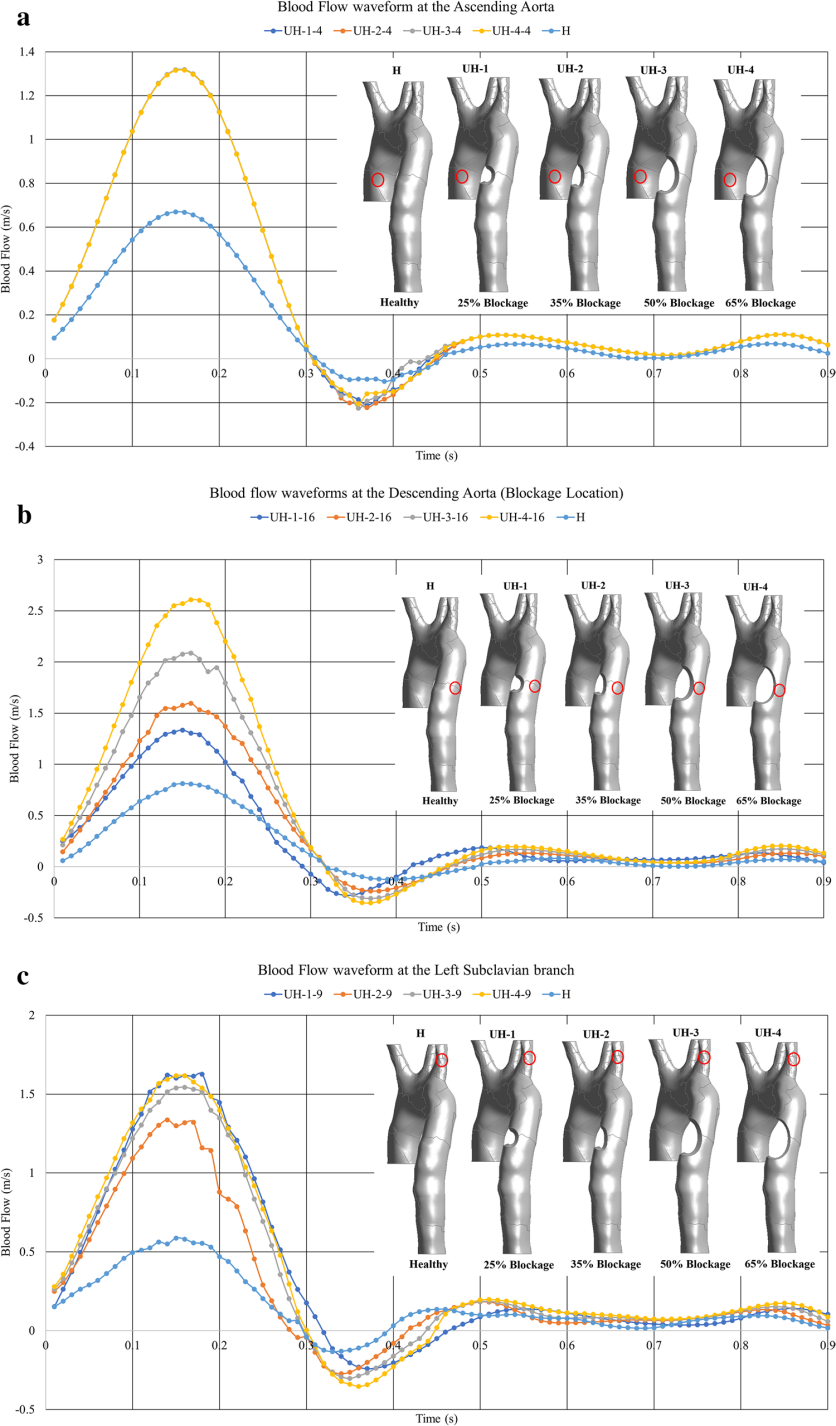
The blood flow waveforms at **a** the ascending aorta; **b** the arterial blockage location; and **c** the left subclavian artery

The velocity differs with the changed lumen diameter, but the volume flow rate stays the same because there is no loss of blood from the system (this differs from aneurysm rupture). Therefore, conservation of mass flow is preserved in this system. Figure 6 shows the blockages’ response to the blood flow velocity at three different locations; the ascending aorta (point 4), blockage location (point 16) and the left subclavian artery (point 9). The change in the blood waveform reflects the unhealthy models responding to stenosis development and growth. Figure 6 indicates that there is greater blood flow velocity associated with the blockage. At the same time, Fig. 6b shows the gradually amplified velocity waveforms with respect to the arterial stenosis growth combined with a clear phase time delay. At the left subclavian artery, the reading in Fig. 6c shows that blockage severity of more than 50% amplifies the peak of the wave to twice that of the healthy wave and tends to convert the flow to a turbulent regime, whereas this amplification factor is less than two for blockages of less than 50% severity.

**Fig. 7:**
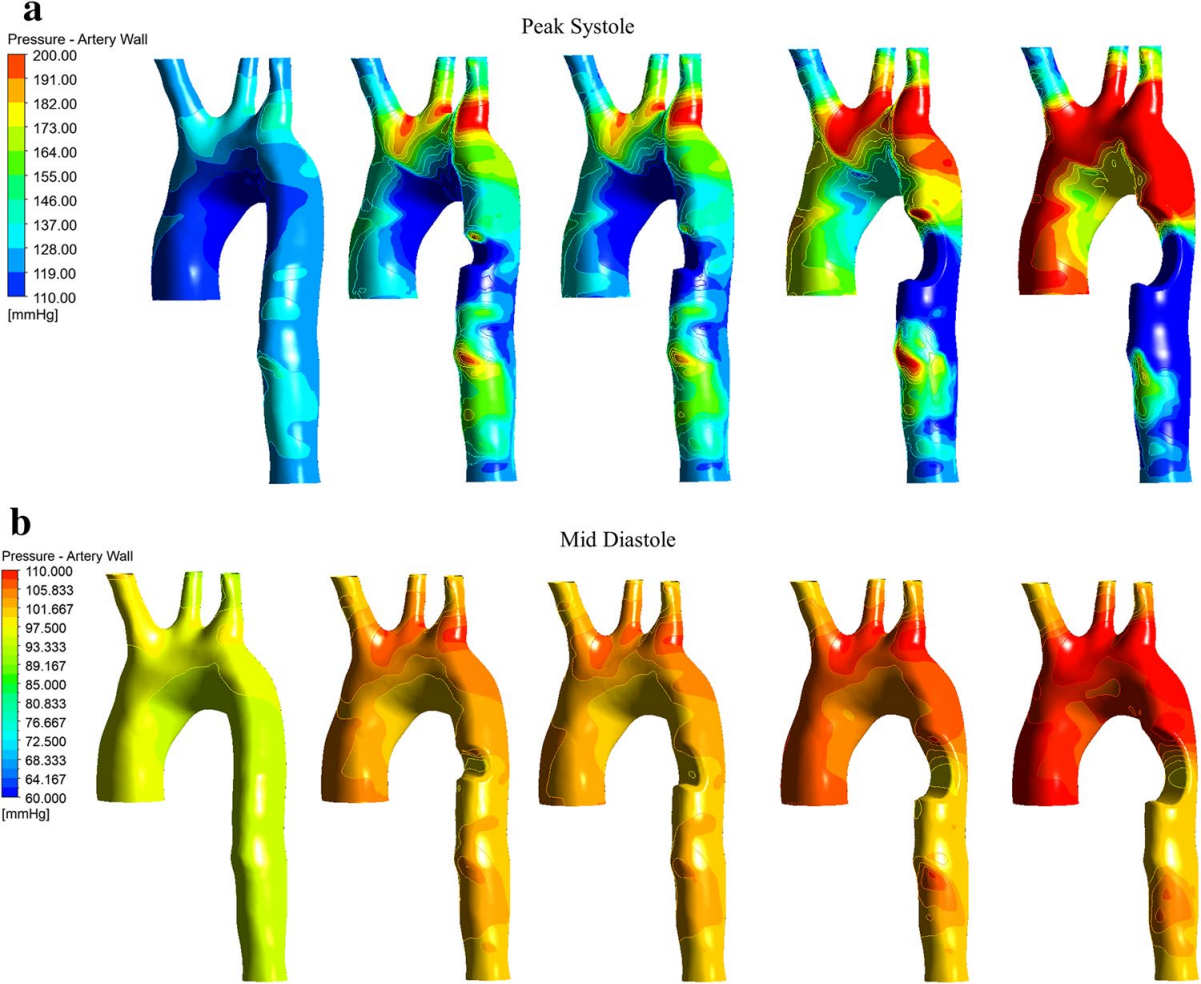
The pressure contours for the healthy and unhealthy models; **a** peak systole and **b** mid-diastole

Figure 7a shows the peak systole pressure contours for healthy (control) and Fig. 7b for the four unhealthy mid-pressure diastole contours. The peak systole contours illustrate a noticeable increase in the high-pressure values at both the arch and the ascending region of the aorta compared to the control model with an increase from 140 to 200 mmHg in unhealthy conditions. At the same time, the mid-diastole pressure contours, illustrated in Fig. 7b, identify a stenosis of more than 50% of the area of the aortic arch; the branch bifurcation intersection is under high-pressure values. This finding confirms what Kamangar et al. [15] described: the impact of stenosis development on the hemodynamic parameters results in high-pressure values correlated to the percentage of the area stenosis combined with a recirculation zone after the injury location. This recirculation zone triggers further progression of arterial blockages such as stenosis in the flow-disturbed area [15] as shown in Fig. 7b.

### Wall shear stress

The WSS is a crucial biomechanical feature linking blood flow to particular changes to the artery wall and the potential for damage, or lesions, associated with cardiovascular disease [36–40]. The value of the WSS is significantly impacted by the flow condition, as described in Eq. (3) below:

5 $$\tau =\mu \sqrt{\left(\frac{\partial {u}_{i}}{\partial {x}_{j}}+\frac{\partial {u}_{j}}{\partial {x}_{i}}\right)}$$

where *τ* is the calculated value of the WSS; *μ* is viscosity; and $\left(\frac{\partial {u}_{i}}{\partial {x}_{j}}+\frac{\partial {u}_{j}}{\partial {x}_{i}}\right)$ is a term described in Tada et al. [40] reflecting the second invariant of the rate of deformation.

Assumptions regarding fluid properties can impact the quality of the model. Where the arterial segment studied has small curvature and high flow, it may be more suitable to analyse the blood using non-Newtonian properties. In this situation, a greater density of red blood cells on the inner radius of the curved vessel corresponds to low WSS [37–40]. Therefore, in this study, we are using the Carreau-Yasuda model showing high values for WSS at the artery wall compared to other studies that used constant viscosity of 0.0035 Pa.s [1].

**Fig. 8:**
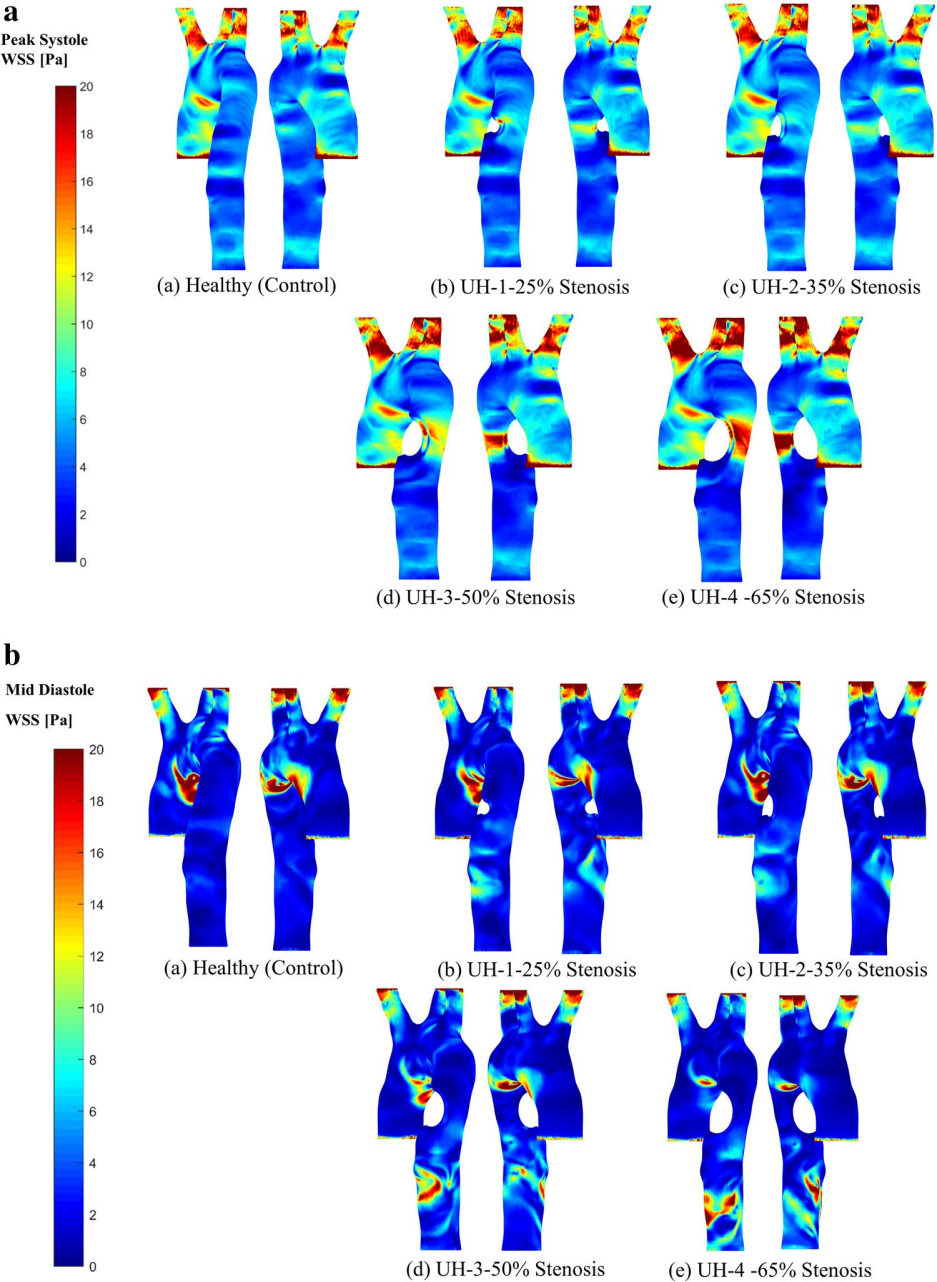
WSS contours for **a** the peak systole; and **b** the mid-diastole for the healthy model and the four unhealthy models

Figure 8a shows the wall shear contours at peak systole, healthy (control) and the four unhealthy aorta models. Wall shear (WSS) contours with a stenosis of 50–65% show high changes to the values at the aorta branch bifurcation. The WSS contours response to the atherosclerosis severity at the blockage location and the bifurcation has no major effect after the disease location compared to the healthy aorta. This finding is observed in the previous study, indicating that the maximum WSS contours can be detected at the bifurcation of unhealthy aorta models [12]. The literature has also indicated that the maximum WSS contours do not occur at the throat of the blockage (stenosis) of the abdominal aorta even if it was stenosed severely [12]. Morris et al. [33] and Stroud et al. [39] found that, while the highest WSS contours in the aortic arch occurred adjacent to its inner wall, these contours differed depending on whether one assumed a steady or a pulsatile flow [36–40]. Valencia et al. [41] found that an artery with atherosclerosis was associated with high WSS contours and pressure at the location of the disease, and the presence of a recirculation zone. During each cardiac cycle, the amount of this recirculating blood could vary, whereas the recirculating zone’s length depended on the degree of stenosis (the disease severity) [41].

Figure 8b shows the WSS contours for the mid-diastole for the control (healthy model) and the four blockage models. For mid-diastole, we found that the high WSS contours appear at the lower part of the aortic arch and have no impact at the stenosis location. We also observed that the high WSS contours start appearing with 50–65% severity at the abdominal aorta compared to the other 25–30% severity. This is because the local WSS naturally presents with oscillations during the cardiac cycle where the maximum contour occurs at the systole peak, while the minimum occurs at the early systole and late diastole. Our results also agreed with Dabagh et al. [12] that the nature of the blockage distribution for asymmetric stenosis along the aorta greatly impacts on the flow pattern through the distribution of WSS contours.

## Discussion

### Time-averaged wall shear stress

In this study, the hemodynamics characteristics using transient pulsatile boundary conditions are investigated in the reconstructed unhealthy aorta model with four descending blockages compared to the control model. The assumption of the transient flow (laminar to turbulent) reflects the fact that endothelial cells are impacted by the fluid’s behaviour, whereby the flow of blood exerts a mechanical force on the artery wall; hence, as previously recommended by [42–46], it is essential to integrate the effect of these forces on the endothelial layer. The laminar flow assumption is recommended based on clinical investigations concerned with cardiovascular disease development in the aorta [46]. Therefore, for aorta solved with a rigid wall, incompressible blood flow and non-Newtonian properties based on the Carreau-Yasuda model, the laminar assumption is acceptable for assessing WSS and TAWSS [46].

Exporting the WSS vectors (100 steps in x, y and z directions) to MATLAB obtains the TAWSS contours for the healthy (control) and four unhealthy models. The TAWSS can evaluate the deviation of WSS vectors on the artery wall while the blockage is developing using the following Eq. (6), which is calculated over a time cycle [12].

6 $$TAWSS=\frac{1}{T}{\int }_{0}^{T}\left|WSS\right|dt$$

**Fig. 9:**
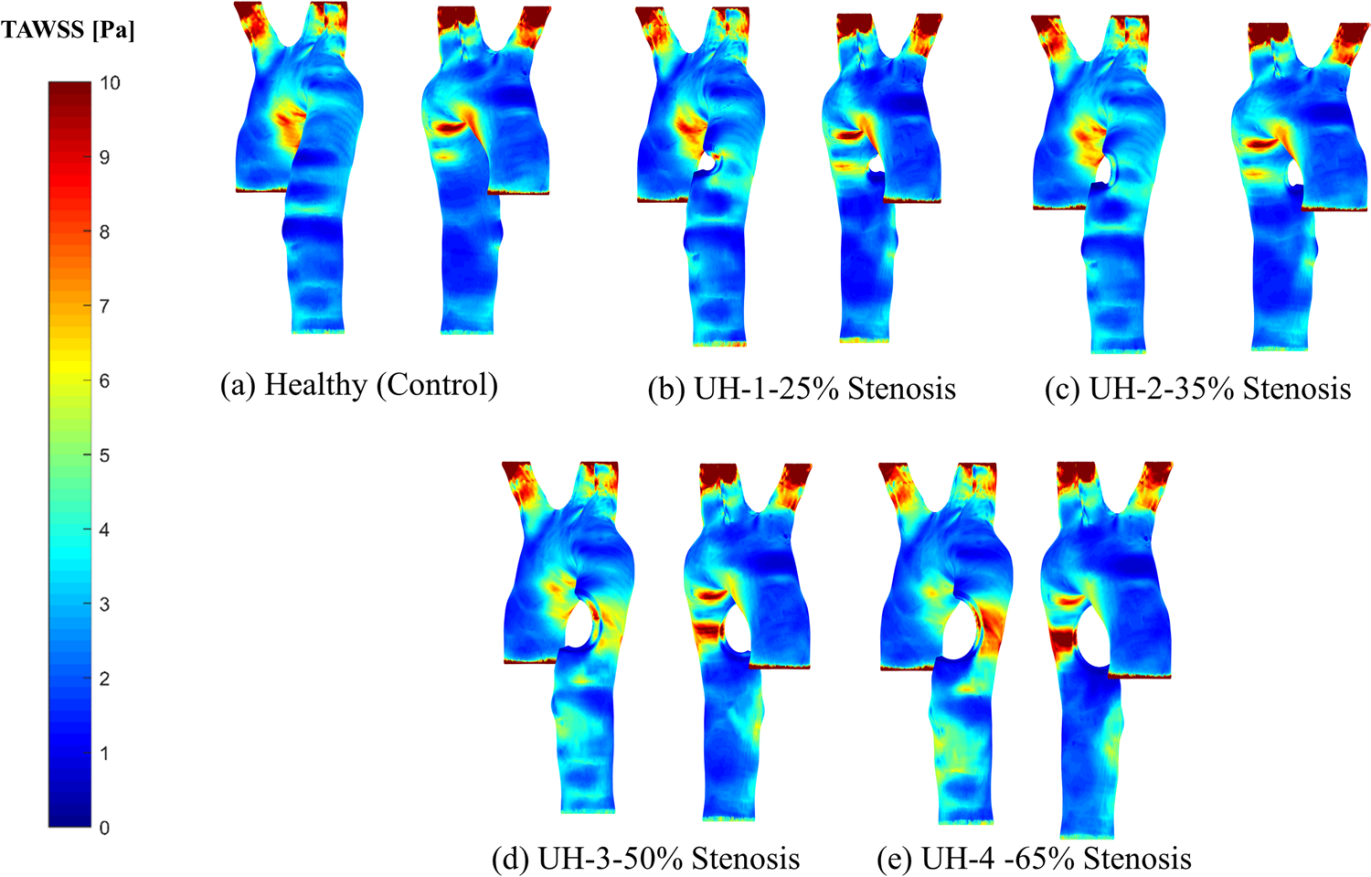
TAWSS for the healthy model and the four unhealthy models: **a** healthy; **b** 25% severity; **c** 35% severity; **d** 50% severity; **e** 65% severity

Figure 9 shows the TAWSS contours for the healthy (controlled model) and the four stenosed models. For the UH-4–65% severity, the TAWSS at the blockage location (descending aorta) attains maximum value. Also, the aortic arch is less affected by the WSS due to the maximum stress at the stenosis location. Low values of TAWSS stimulate proatherogenic endothelial phenotype [16], and cause wall endothelial cells to suffer perturbation at the zones where the sudden WSS contours differ from the main blood flow pattern for a sizeable portion of the cardiac cycle. These manifest as zones with high OSI contours as shown in Fig. 9.

### Oscillation shear index

Another hemodynamic parameter related to the flow oscillation is the oscillation shear index (OSI). The OSI can be used to define the WSS oscillations’ amplitude at the arterial wall which will be used to see the blockage growth using,

7 $$OSI=\frac{1}{2}\left(1-\frac{\left|{\int }_{0}^{T}\overrightarrow{{\tau }_{w}}dt\right|}{{\int }_{0}^{T}\left|\overrightarrow{{\tau }_{w}}\right|dt}\right)$$

**Fig. 10:**
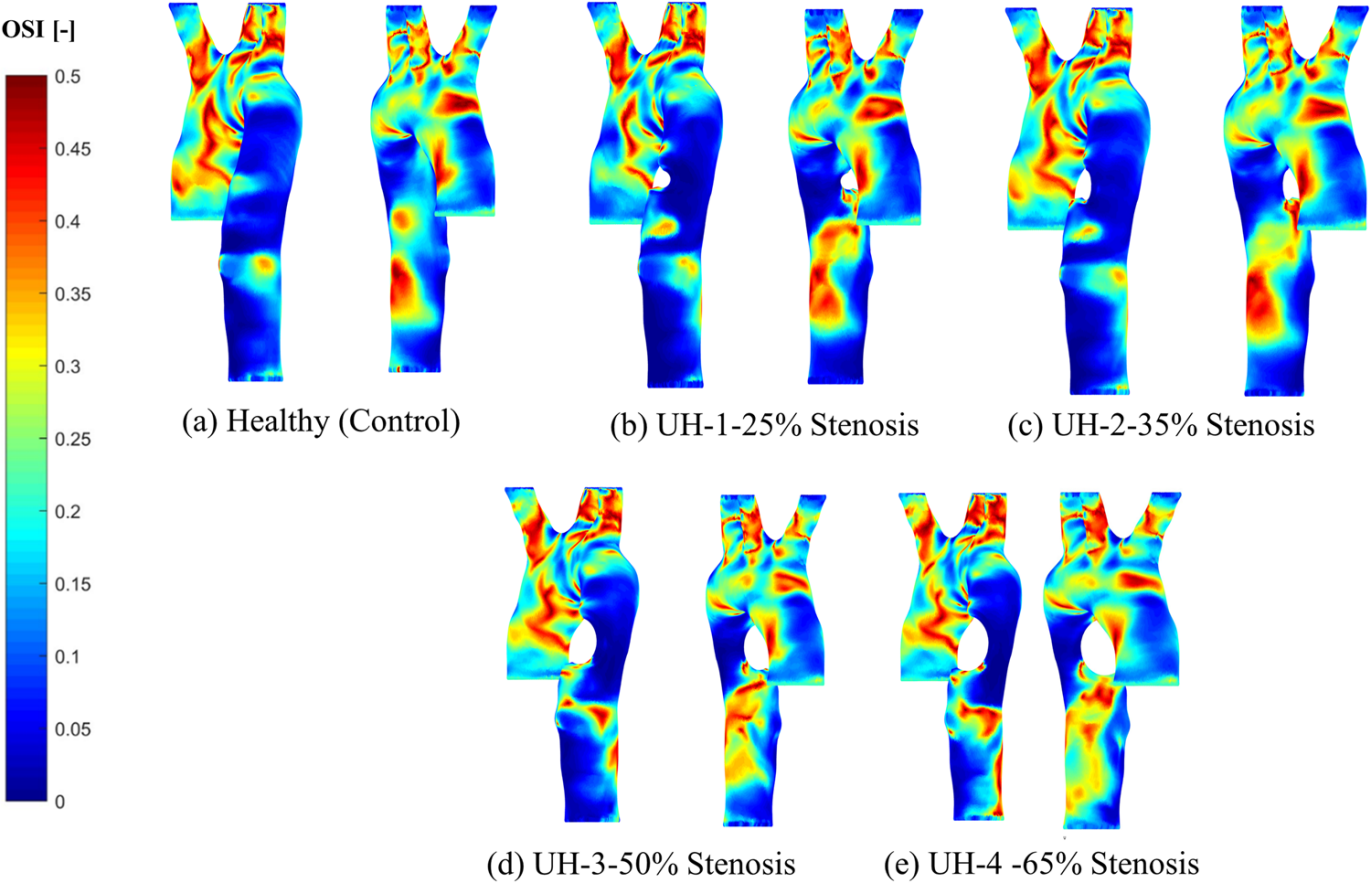
The OSI for the healthy model and the four unhealthy models: **a** healthy; **b** 25% severity; **c** 30% severity; **d** 50% severity; **e** 65% severity

OSI has a range between 0 (for constant WSS vectors) and 0.5 (for turbulent unsteady WSS vectors), where 0.5 reflects solely high oscillatory flow [18]. Where OSI is high, this causes disruption to the endothelial structure and atherogenesis; consequently, it is important to identify these zones. OSI contours never exceeded 0.2, considering the healthy and unhealthy models with the arterial wall blockage development. High OSI contours are detected at the bifurcations, reflecting low oscillatory wall shear stress development at the disease site. Figure 10 shows the healthy model with normal distribution with OSI close to 0.4 at the aortic arch, compared to the arterial blockage’s development at the 65% severity in case UH-4 exceeds 0.5 before and after the blockage location.

In our study, we observed low WSS contours along with high OSI contours at the same region of stenosis growth at the descending aorta; however, the OSI contours cannot be relied upon where there is turbulent flow at that location. Consequently, the RRT, which incorporates OSI and TAWSS, can be used to examine and analyse the areas with low WSS to determine the risk of the arterial blockage [20].

### Relative residence time

RRT, a function of OSI and TAWSS, is proportional to the time blood particles spend near the artery wall. This proportionality is significant, as the results are normalized. The OSI acts to modify the effect of the TAWSS on the RRT at a particular location along the endothelium, as illustrated in Eq. (8) below:

8 $$RRT=\frac{1}{(1-2\times OSI)\times TAWSS}$$

**Fig. 11:**
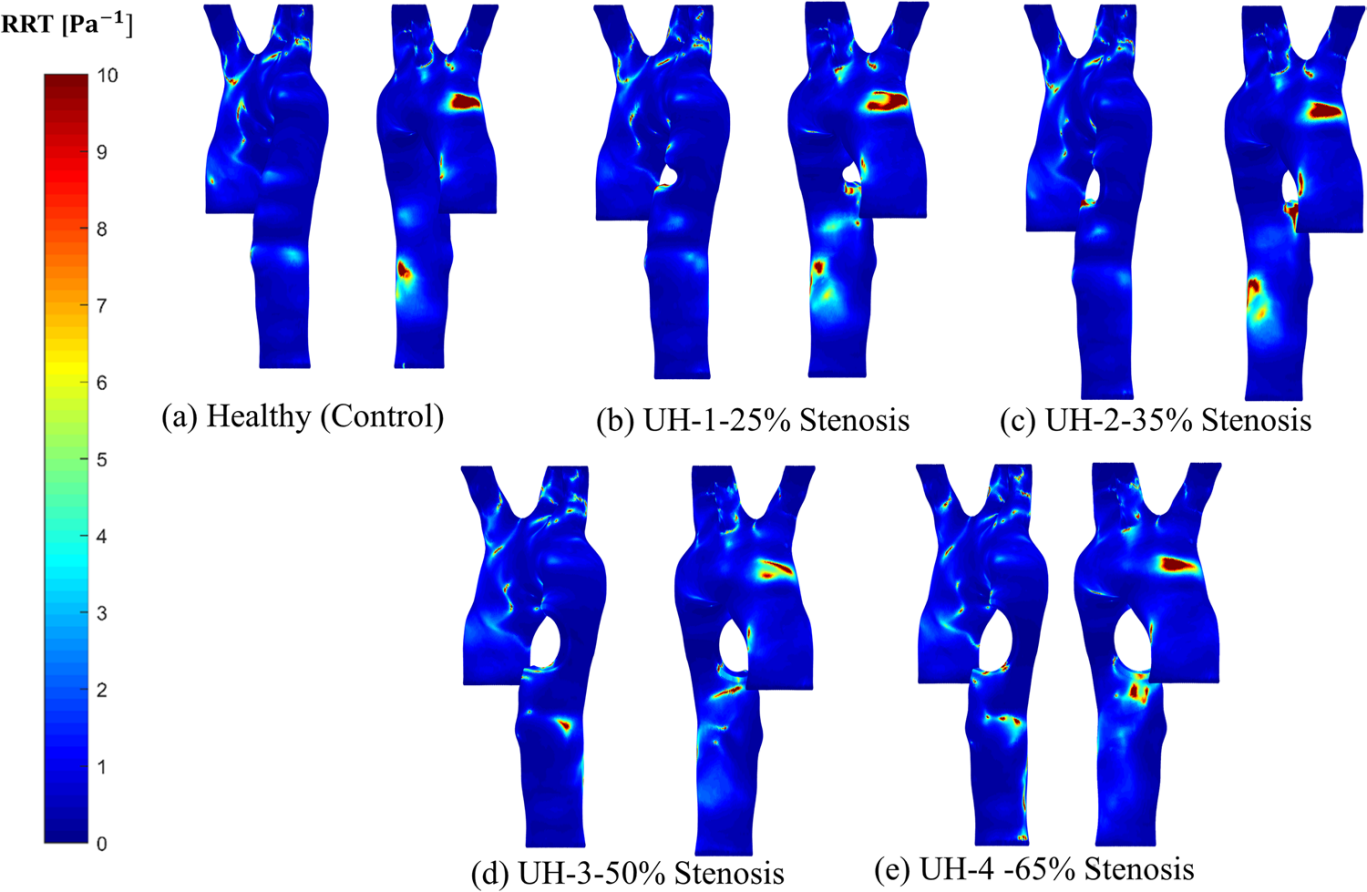
The RRT contours for the healthy model and the four unhealthy models: **a** healthy; **b** 25% severity; **c** 30% severity; **d** 50% severity; **e** 65% severity

RRT contours for the controlled healthy model and the four unhealthy models with blockages are represented in Fig. 11. The healthy model with the invasive boundary conditions applied and using Carreau-Yasuda non-Newtonian model shows high RRT contours near the bifurcation region of the aortic arch and the abdominal aorta, compared to other segments of the aorta. At the descending aorta, it was found that the 65% blockages have a more significant influence on the RRT contours. This influence starts from the 25% blockage and the surface area of the RRT contours starts increasing with the increase of the blockage growth.

Figure 11 shows the RRT contours for the healthy model and the four severe blockages (25%, 35%, 50% and 65%). There is no significant effect on the RRT contours for the 25–50% blockages whereas the RRT contours are influenced by the higher degree of blockage at the disease location, as we can see with the results for the 50–65% blockages compared to the healthy condition. However, we found the high contours for RRT at 50–65% severity disappeared after the disease’s location due to the high TAWSS generated at that location, as shown in Fig. 9.

**Fig. 12:**
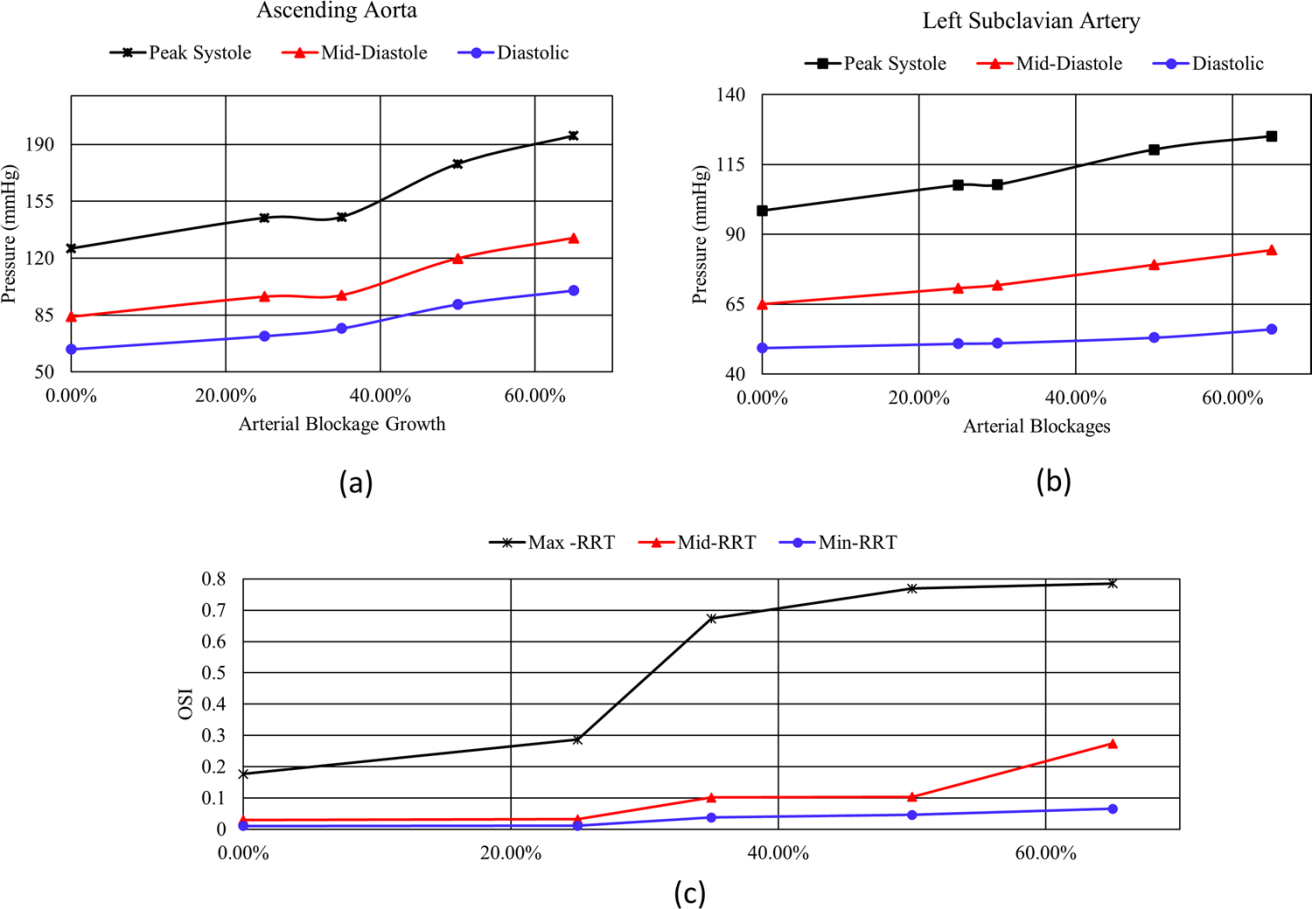
A comparison between the systole, mid-diastole and diastolic pressure values at **a** the ascending aorta; **b** left subclavian artery; and **c** along the RRT (max, mid and min) values with respect to the arterial blockage growth

The results of the blockage growth development at the ascending aorta are correlated to the systole pressure, the diastole pressure and the mid-diastole pressure to the maximum, minimum and medium RRT. The correlation shows that an increase in pressure values at ascending aorta and left subclavian artery is combined with an increase in the RRT which is based on the OSI with the maximum value of 0.5 and TAWSS growth along the aorta geometry as shown in Fig. 12. The RRT explains the behaviour change in the blockage development on the blood flow conditions using non-Newtonian blood flow (Carreau-Yasuda model). This model illustrates a high value for WSS at the artery wall compared to other studies that used Newtonian blood assumptions.

## Conclusion

This is a computational study based on a specific healthy patient treated as a control model to investigate the P waveforms, WSS, TAWSS, OSI and RRT for four models created based on the control model. The results show a strong relationship between the hemodynamics parameters (WSS, TAWSS, OSI) and the left subclavian artery’s pressure waveform. The waveforms are correlated to the severity of the four descending aorta blockages, representing 25%, 35%, 50% and 65% stenosis, using the Carreau-Yasuda model. The results show that high RRT values develop with respect to an increase of the pressure contours during early systole, mid-systole and diastole at both the ascending aorta and the left subclavian artery. The main reason behind that is that the molecular viscosity presented in the Carreau-Yasuda model elevates the OSI which emerges with high RRT to identify possible growth of stenosis due to atherosclerotic plaque at the descending aorta. This finding regarding the reading of pressure values could be transferred to the brachial artery in future studies.
